# Relationships Between Gastrocnemius Medialis Muscle Architecture, Vertical Stiffness, and Vertical Jump Performance in Track and Field Athletes

**DOI:** 10.3390/jfmk11040407

**Published:** 2026-10-05

**Authors:** Wenjing Tang, Zhenyu Peng, Heng Lu, Armin H. Paravlić, Ran Wang

**Affiliations:** 1 School of Athletic performance, Shanghai University of Sport, Shanghai 200438, China; 2211111002@sus.edu.cn (W.T.); luheng@sus.edu.cn (H.L.); 2 School of Computer Science and Technology, Donghua University, Shanghai 201620, China; 19857105089@163.com; 3 Faculty of Sport, University of Ljubljana, 1000 Ljubljana, Slovenia; armin.paravlic@fsp.uni-lj.si; 4 Science and Research Centre Koper, Institute for Kinesiology Research, 6000 Koper, Slovenia; 5 Faculty of Sports Studies, Masaryk University, 625 00 Brno, Czech Republic

**Keywords:** countermovement jump, squat jump, muscle thickness, pennation angle, vertical stiffness, track and field athletes

## Abstract

**Background and Objectives:** Gastrocnemius medialis (GM) muscle architecture and vertical stiffness have been associated with vertical jump performance, but their relationships across jumping tasks with different stretch-shortening cycle (SSC) demands have not been well characterized. This exploratory study aimed to characterize the relationships of GM muscle architecture and Kvert with vertical jump performance in collegiate track and field athletes. **Methods:** Thirteen athletes (age: 21.62 ± 2.60 years; height: 1.82 ± 0.06 m; body mass: 74.72 ± 8.72 kg) completed squat jump (SJ), countermovement jump (CMJ), and 30-cm drop jump (DJ-30) tests. GM muscle thickness (MT), pennation angle (PA), and estimated fascicle length (FL) were assessed using ultrasonography, and vertical stiffness (Kvert) was calculated from the CMJ. **Results:** Pearson correlation analyses identified positive correlations between GM PA and SJ peak force (*r* = 0.66, *p* = 0.01) and CMJ peak force (*r* = 0.56, *p* = 0.04), peak power (*r* = 0.62, *p* = 0.02), and jump height (*r* = 0.55, *p* = 0.04), while GM MT was positively correlated with CMJ peak velocity (*r* = 0.56, *p* = 0.04) and relative peak power (*r* = 0.62, *p* = 0.02). Kvert was also positively correlated with CMJ relative peak force (*r* = 0.62, *p* = 0.02). In contrast, no GM muscle architecture variables were significantly correlated with DJ-30 performance. However, none of these correlations remained statistically significant after adjustment for multiple comparisons. **Conclusions:** The findings suggest that GM PA and MT may be more closely related to performance in jump tasks with no or slower SSC demands (SJ and CMJ) than to fast SSC tasks (DJ-30). These findings therefore provide preliminary information for further investigation of how different aspects of GM architecture relate to specific components of jumping performance, although confirmation in larger samples is still needed.

## 1. Introduction

Physical profiling of athletes is often performed to identify influential characteristics of key performance indicators of the sport. Across the speed/power-based sprint and jump disciplines, acceleration ability, maximal sprint velocity, approach velocity, and take-off velocity are considered key determinants of competitive performance in track and field athletes [1]. These performance outcomes are underpinned by neuromuscular qualities such as muscular strength and power, neuromuscular efficiency, reactive strength, and lower-limb stiffness [1,2,3]. Related to this, countermovement jumps (CMJ), squat jumps (SJ) and drop jumps (DJ) are commonly used in physical profiling to assess lower-limb strength and power, stiffness-related characteristics, and reactive strength [2,3,4,5].

Previous studies have sought to identify the neuromuscular characteristics that underpin performance in jumping tasks. Among these, muscle architecture is considered a key determinant of jumping performance, as the arrangement of muscle fibers within a muscle influences the force-velocity and force-length characteristics of muscle fascicles [6]. Longer fascicles may facilitate force production at higher shortening velocities and across a greater range of muscle lengths, whereas greater muscle thickness (MT) and pennation angle (PA) are generally associated with a greater force-producing capacity [6,7]. One of the most consistent findings to date is that muscle size (i.e., MT and anatomical cross-sectional area), PA, and fascicle length (FL) of the vastus lateralis (VL) and lateral gastrocnemius (LG) are associated with key performance variables during SJ, CMJ, and DJ, including jump height, peak power, mean power, peak force, and peak velocity [8,9,10,11,12,13,14,15,16,17]. 

The triceps surae muscles, comprising the soleus and the medial and lateral gastrocnemii, make substantial contributions to ankle joint power generation during vertical jumping [18,19]. However, despite the growing evidence linking calf muscle architecture with jumping performance, most previous research has focused on the LG [8,9,10,11,13,14], whereas the gastrocnemius medialis (GM) has received comparatively limited attention. Importantly, the GM and LG are not functionally interchangeable, as they differ in architectural characteristics, activation patterns, and fascicle–tendon behavior, which may contribute to distinct neuromechanical roles during locomotion and force production [20,21,22]. Findings from the few available studies suggest that the relationship between GM architecture and jump performance may be task specific. For example, Robshaw et al. [23] reported that in male academy soccer players, GM FL correlated inversely with vertical CMJ performance but positively with horizontal CMJ performance, whereas GM PA showed the opposite pattern. In the same study, different correlation patterns were observed in age- and sex-matched control participants [23]. Similarly, previous work in boys showed that GM and VL MT explained 38.5–55.8% of the variance in peak force, impulse, and power variables during maximal rebound jumping in young boys, whereas the explained variance for jump height and velocity was considerably lower [24]. These findings suggest that relationships between GM architecture and jumping performance may vary according to task demands, movement direction, the selected performance variable, and population characteristics. More importantly, muscle architecture is responsive to sport- and training-specific loading [25,26,27], further limiting direct extrapolation of findings across athletic populations. Therefore, whether GM muscle architecture is systematically related to performance across different vertical jumping tasks in track-and-field athletes remains unclear. 

Beyond muscle architectural characteristics, lower-limb stiffness provides another perspective on the mechanical demands of jumping performance [3,28]. In stretch-shortening cycle (SSC) activities, lower-limb stiffness reflects the relationship between force production and displacement and is commonly used to describe its mechanical response of the lower extremity system during dynamic movements [3,29,30,31]. Vertical stiffness (Kvert), derived from the spring–mass model, provides a task-level index of this force–displacement behavior and is calculated as the ratio of peak vertical ground reaction force to vertical displacement of the body’s centre of mass (COM) [30]. Previous studies have reported relationships between calculated lower-limb stiffness measures and both muscle architecture and jumping performance. For example, Secomb et al. [13] reported that Kvert was related to CMJ peak force and jump height, as well as to LG PA, in elite male surfing athletes. Similarly, eccentric leg stiffness derived from the CMJ showed moderate-to-large relationships with LG MT and LG FL, and a large relationship with IMTP PF in adolescent athletes [14]. These findings suggest that lower-limb stiffness may provide a complementary mechanical perspective to muscle architectural characteristics when examining the factors associated with jumping performance. However, Kvert has also been shown to vary according to training background [32], and its relationships with GM muscle architecture and jumping performance still remain unclear in track-and-field athletes. 

Based on the current evidence, the present exploratory study aimed to characterize the relationships among GM muscle architecture, Kvert, and vertical jump performance in collegiate track-and-field athletes. Clarifying these relationships may provide a more complete understanding of the neuromuscular and mechanical characteristics associated with jumping performance and may inform athlete profiling and training monitoring in track and field.

## 2. Materials and Methods

### 2.1. Study Design

This exploratory cross-sectional study involved two testing sessions separated by approximately 24 h. Data collection was conducted during the pre-season preparatory phase of the athletes’ annual training cycle, during which athletes continued their habitual track and field training, including resistance, sprint, plyometric, and other power-oriented training. During the first session, anthropometric measurements and GM muscle architecture were assessed using ultrasonography. During the second session, vertical jump performance was assessed using the SJ, CMJ, and 30 cm DJ (DJ-30). Jump testing was performed after the ultrasound assessment to avoid potential acute alterations in muscle architecture associated with exercise-induced muscle damage and edema following jumping exercise. During the first session, measurements were obtained in the following order: height, body mass, GM MT, and PA. The three jump tasks were selected to represent different SSC demands [9]. Athletes were instructed to refrain from intense or unaccustomed exercise for at least 48 h and all exercise for at least 24 h before testing. They were also instructed to avoid caffeine and alcohol for at least 12 h before testing and to maintain their habitual diet and hydration status throughout the testing period.

### 2.2. Athletes

Thirteen collegiate track and field athletes with at least 5 years of structured training experience voluntarily participated in this study, including 11 males and 2 females. Athletes represented multiple track and field disciplines, and their primary events and personal best performances are presented in Appendix A.2. Athlete characteristics are presented in Table 1. Eligibility criteria were: (a) active participation in competitive track and field training and competition; (b) age ≥ 18 years; and (c) no current musculoskeletal injury or medical condition that could affect testing performance. All athletes had engaged in regular resistance training for at least 6 months (≥2 sessions per week). Before data collection, athletes were familiarized with all testing procedures and practiced each task until they demonstrated appropriate technique as judged by the researchers. No technical feedback was provided during the formal testing sessions. The study protocol was approved by the university institutional review board and conducted in accordance with the Declaration of Helsinki. All subjects were informed of the study procedures and potential risks and provided written informed consent before participation.

### 2.3. Procedures

#### 2.3.1. Ultrasound

Muscle structure of the GM was assessed using real-time B-Mode ultrasonography (LOGIQ e, GE HealthCare, Wuxi, China) with a 7.5-MHz linear array probe. A water-soluble gel was applied between the probe and the skin to ensure adequate acoustic coupling, and minimal transducer pressure was applied to avoid deformation of the underlying muscle tissue. Athletes were positioned in a relaxed prone position on an examination table, with the knee fully extended and the ankle secured at 0° (neutral position). Measurements were obtained from the dominant leg (defined as athlete’s self-reported preferred kicking leg [33]) at 30% of the distance from the popliteal crease to the center of the lateral malleolus [34]. Ultrasound images were acquired in the longitudinal plane, with the transducer oriented parallel to the GM fascicles and perpendicular to the skin surface, allowing the fascicles and aponeurosis to be visualized along their longitudinal axis.

**Figure 1:**
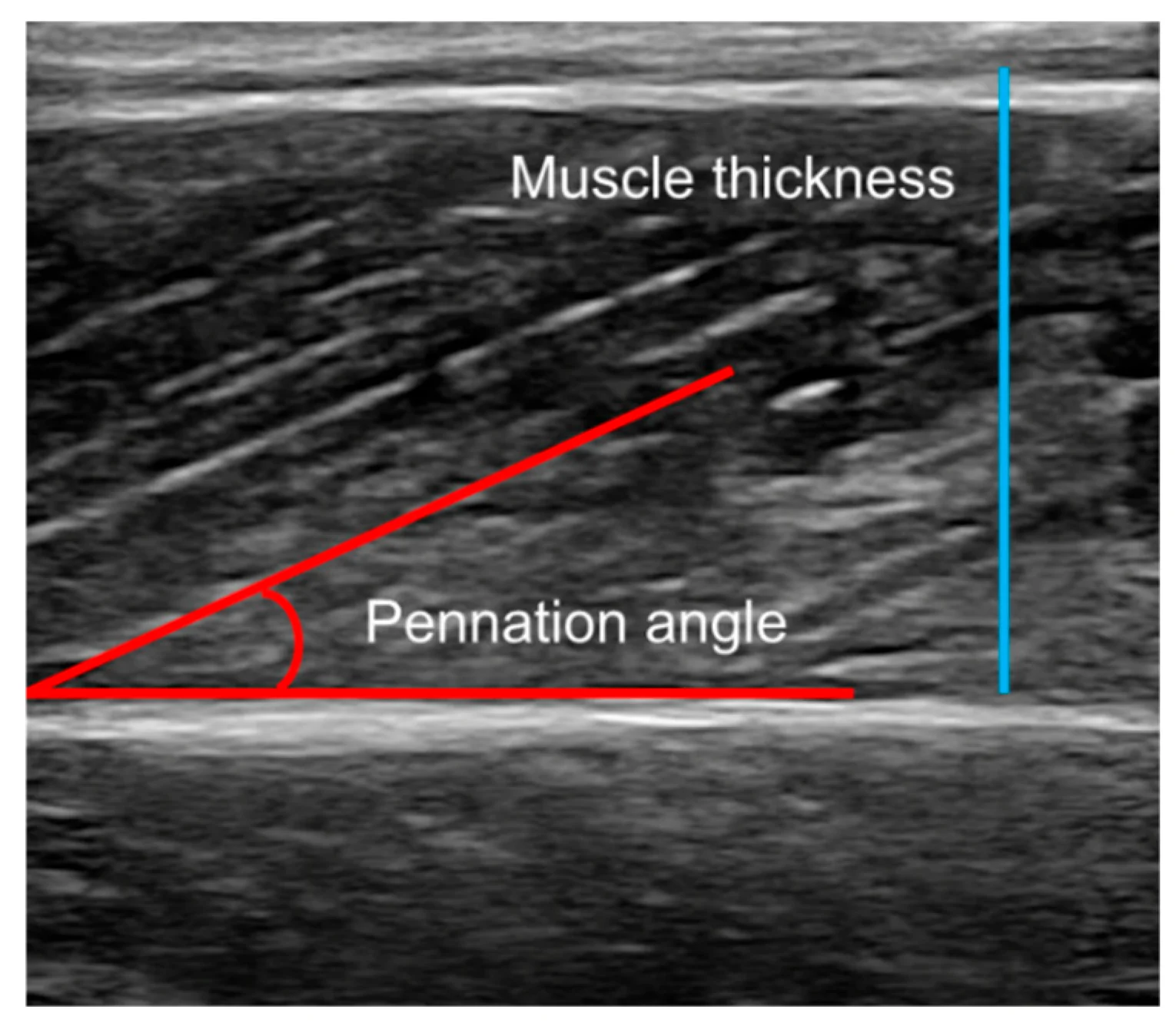
Sagittal ultrasound images of the gastrocnemius medialis.

Ultrasound images were analyzed using ImageJ software (version 1.54r, 25 September 2025) (National Institutes of Health, Bethesda, MD, USA). MT was defined as the perpendicular distance between the superficial and deep aponeuroses and was measured at the predefined measurement site (Figure 1). PA was defined as the angle between the muscle fascicles and the deep aponeurosis (Figure 1). Three ultrasound images were acquired sequentially at the same predefined measurement site, with the transducer lifted from the skin and repositioned between image acquisitions. MT and PA were independently measured on each image, and FL was subsequently estimated from the measured MT and PA using the equation FL = MT/sin (PA). The mean values across the three images were used for statistical analysis. All ultrasound measurements were performed by the same experienced technician to minimize inter-rater and operator-related variability.

Muscle thickness (MT) was measured as the perpendicular distance between the superficial and deep aponeuroses, and pennation angle (PA) was measured as the angle between the muscle fascicles and the deep aponeurosis.

#### 2.3.2. Vertical Jump Testing

Before vertical jump testing, all subjects performed a standardized warm-up consisting of moderate-intensity cycle ergometry and dynamic lower-body stretches. Static stretching was not included because it may impair subsequent power production and alter musculotendinous function [10]. The SJ, CMJ, and DJ-30 were performed in a randomized and counterbalanced order. Each jump type was performed in two nonconsecutive sets of three maximal-effort trials. Subjects completed one set of each jump type before repeating the sequence for the second set, with 3 min of rest between sets to allow sufficient recovery and minimize potential order effects. Throughout the testing, subjects kept their hands on their hips to minimize the contribution of upper-body movements. All jumps were performed on a calibrated force plate (KWFP5060-5K; kunwei Tech, Changzhou, China) with a sampling at 1000 Hz. 

For the CMJ, athletes began from an upright standing position, performed a self-selected countermovement to a preferred depth, and then jumped vertically as high as possible. They were verbally instructed to maximize jump height while maintaining their self-selected countermovement depth. After landing, subjects returned to an upright standing position and rested for 30 s before the next trial. For the SJ, subjects adopted a predetermined starting position, maintained this position for 2 s, and then jumped vertically as high as possible without a preceding countermovement. They were verbally instructed to remain stationary during the 2-s stabilization period and then jump as high as possible without a preparatory downward movement. The SJ starting position was standardized based on each athlete’s self-selected countermovement depth recorded during familiarization. The force-time trace of each trial was visually inspected to identify any countermovement before take-off; trials showing a discernible countermovement were excluded and repeated. 

Bilateral DJs were performed from a 30-cm box. The 30-cm drop height was selected as a standardized testing condition rather than as an individualized optimal drop height. This choice was informed by previous research demonstrating the use of approximately 30 cm in well-trained athletes from sprinting and jumping disciplines [35], as well as by studies that have used 30-cm drop jumps in elite or high-level track and field sprinters and jumpers [36,37]. A fixed height was used to ensure a consistent and reproducible SSC task across athletes from different track and field disciplines. An attempt was only deemed acceptable if contact time was <250 ms [38]. Upon landing on the force platform, subjects were instructed to jump vertically as high as possible while minimizing ground contact time. The reactive strength index (RSI) was calculated as jump height divided by ground contact time, with jump height determined from flight time [39]. For the CMJ and SJ, the trial with the greatest jump height was selected for subsequent analysis of all jump-performance variables. For the DJ, the trial with the highest RSI was selected for analysis of all DJ variables.

The jump tests provided measures of peak force (PF), relative peak force (rPF), peak power (PP), relative peak power (rPP), peak velocity (PV), and jump height across the CMJ, SJ, and DJ-30. Ground contact time (GCT) was additionally obtained from the DJ-30. Kvert was calculated from CMJ as Fpeak/ΔL, where Fpeak represents the peak ground reaction force and ΔL represents the vertical displacement of the center of mass [3]. All jump performance variables were calculated using Microsoft Excel 2021 (Microsoft Corporation, Redmond, WA, USA). Detailed descriptions of the calculation procedures are available elsewhere [40,41].

### 2.4. Statistical Analyses

Because this was an exploratory cross-sectional study, no formal a priori sample-size calculation was performed before data collection. Given the final sample of 13 athletes, a sensitivity power analysis was conducted using G*Power 3.1.9.7 (Heinrich Heine University Düsseldorf, Düsseldorf, Germany) with a two-tailed α = 0.05 and ρH0 = 0. The analysis indicated that the sample provided 80% power to detect a population correlation of approximately |*r*| ≥ 0.68.

Descriptive statistics for all jump performance variables and Kvert are presented as mean ± standard deviation (SD) in Table 2. Data normality was assessed using the Shapiro–Wilk test. Pearson correlations were used to examine the relationships among GM muscle architecture, Kvert, and jump performance. The coefficient of determination (*r*^2^) was calculated as an index of shared variance, and 95% confidence intervals (CIs) were calculated for all correlation coefficients. To control the false discovery rate (FDR) arising from multiple comparisons, the Benjamini–Hochberg procedure was applied to all *p*-values from the primary Pearson correlation analyses. Both unadjusted *p*-values and FDR-adjusted *q*-values were reported, with *q* < 0.05 considered statistically significant. As a sensitivity analysis, partial correlations controlling for body mass, sex, or both were performed for correlations with an unadjusted *p* < 0.05 in the primary analysis. Additionally, reliability was assessed using ICC (3,1) (two-way mixed-effects model, absolute agreement), with a single measurement for the ultrasound variables and a single trial for the jump variables. Absolute reliability was assessed using the coefficient of variation (CV%), calculated for each athlete as the within-athlete standard deviation across repeated measurements divided by the corresponding participant means, multiplied by 100%. All statistical analyses were performed using a statistical analysis package (SPSS, version 27.0; IBM, Chicago, IL, USA).

## 3. Results

### 3.1. Descriptive Statistics and Within-Session Reliability

GM MT, PA, and estimated FL were 2.09 ± 0.17 cm, 18.03 ± 1.33°, and 6.67 ± 0.59 cm, respectively. The within-session reliability of GM muscle architecture variables was high, with ICC values of 0.98, 0.96, and 0.87 and CV values of 1.56%, 1.87%, and 3.35% for MT, PA, and estimated FL, respectively. Descriptive statistics and within-session reliability estimate for jump performance variables obtained from the CMJ, SJ, and DJ-30 are presented in Table 2. The ICC values ranged from 0.83 to 0.99, and CV values ranged from 1.20% to 6.27% across all jump variables. 

### 3.2. Correlations Between GM Muscle Architecture, Kvert, and Jump Performance

Pearson correlation analyses identified seven correlations with an unadjusted *p* < 0.05 among GM muscle architecture, Kvert, and jump performance variables (Table 3). Specifically, GM PA was positively correlated with SJ PF (*r* = 0.66, *p* = 0.01), CMJ PF (*r* = 0.56, *p* = 0.04), CMJ PP (*r* = 0.62, *p* = 0.02), and CMJ height (*r* = 0.55, *p* = 0.04). GM MT was positively correlated with CMJ PV (*r* = 0.56, *p* = 0.04) and rPP (*r* = 0.62, *p* = 0.02). In addition, Kvert was positively correlated with CMJ rPF (*r* = 0.62, *p* = 0.02). The corresponding coefficients of determination (*r*^2^) ranged from 30.3% to 43.6%. However, none of these correlations remained statistically significant after Benjamini–Hochberg FDR correction, with all *q*-values = 0.27. No correlations were identified between estimated FL and any jump performance variable (all unadjusted *p* > 0.05; Appendix A Table A1).

### 3.3. Sensitivity Analyses of Covariate-Adjusted Partial Correlations

Sensitivity analyses using partial correlations showed that, after adjustment for body mass, only the correlations of GM PA with SJ PF (*r* = 0.59, *p* = 0.04) and GM MT with CMJ rPP (*r* = 0.62, *p* = 0.03) remained statistically significant (Table 4). After adjustment for sex alone or for body mass and sex simultaneously, none of the correlations remained statistically significant. In addition, the association between Kvert and CMJ relative PF was not statistically significant after adjustment for body mass (*r* = 0.29, *p* = 0.35), sex (*r* = 0.32, *p* = 0.29), or both (*r* = 0.25, *p* = 0.44).

## 4. Discussion

The present exploratory study aimed to characterize the relationships among GM muscle architecture, Kvert, and vertical jump performance in collegiate track-and-field athletes. The main findings were that (i) GM PA was positively correlated with several CMJ and SJ performance variables, whereas GM MT was positively correlated with selected CMJ variables; and (ii) Kvert showed a large positive correlation with relative PF during the CMJ. However, none of these associations remained statistically significant after correction for multiple comparisons and should therefore be regarded as exploratory rather than confirmatory findings.

To the best of our knowledge, this is the first study to report the relationships between GM muscle architecture and jump performance in track and field athletes. Consistent with our findings, a recent study by Robshaw et al. [23] also reported positive associations between GM PA and vertical CMJ performance in adolescent soccer players. A larger PA is thought to represent a greater number of sarcomeres arranged in parallel, which may contribute to a greater physiological cross-sectional area of the muscle fibers [6]. In return, this structural arrangement provides a greater amount of contractile tissue attaching to the aponeurosis, thereby potentially increasing the muscle’s capacity to generate force [27]. Although muscles with a greater PA exhibit greater force attenuation at high contraction velocities according to the force–velocity relationship [42,43], this apparent “force loss” remains relatively small (e.g., ~6% at 20°) and is insufficient to offset the force-generating advantage conferred by fiber packing within a pennate muscle architecture [44]. These architectural characteristics therefore provide a plausible physiological basis for the relatively large positive correlations observed between PA and force- and power-related outcomes during the CMJ and SJ. However, given the exploratory design, small sample size, and multiple comparisons, these relationships require confirmation in larger samples. 

Additionally, the positive correlations between GM MT and CMJ rPP and PV in the present study are consistent with previous findings [24,45]. For example, Radnor et al. [24] reported that GM and VL MT explained 38.5% to 55.8% of the variance in PF, impulse, and power-related variables during maximal rebound jumping in young boys. Similarly, Lee et al. [45] found that calf MT was an important structural determinant of anaerobic power performance, with GM MT significantly correlated with PP. Previous evidence suggests that greater MT may reflect a greater number of actin and myosin filaments within the muscle, thereby allowing greater force production [13]. This may increase the potential for cross-bridge formation within the muscle fibers [13], such that greater force output can contribute to higher power and velocity during explosive movements such as the CMJ. Furthermore, Earp et al. [9] reported that LG MT was the strongest predictor of absolute power across different types of vertical jumps, including the SJ, CMJ, and DJ. Collectively, previous evidence and the relatively large correlations observed in the present study suggest that triceps surae MT and PA may warrant further investigation as structural characteristics related to lower-body force and power performance. It should also be noted that several associations involving absolute force or power were stronger than those observed for body-mass-normalized outcomes, indicating that body size may have contributed to some of the observed relationships. In sensitivity analyses, adjustment for body mass attenuated most of the unadjusted associations. However, the associations of GM PA with SJ PF (*r* = 0.59, *p* = 0.04) and GM MT with CMJ *r*PP (*r* = 0.62, *p* = 0.03) remained statistically significant. These findings suggest that these two associations were not entirely explained by body mass, although they should still be considered preliminary because neither survived FDR correction. Future longitudinal studies are needed to determine whether changes in GM MT and PA are accompanied by corresponding changes in CMJ and SJ performance.

Unexpectedly, the present study found no significant relationships between GM FL and any jump performance variable. This may reflect the task-specific role of FL in human movement. Although FL is related to muscle shortening velocity, its functional relevance may depend on movement direction and mechanical demands [23]. Previous studies have reported inconsistent associations between FL and jump performance, with negative relationships observed in vertical jumping but positive relationships in horizontal movements [23,46]. This difference may be related to the relatively small FL changes during vertical jumping, where much of the muscle-tendon unit length change occurs at the tendon while the fascicles operate near their optimal length and shorten relatively slowly [19,47,48,49]. Therefore, although longer fascicles theoretically increase maximal shortening velocity by accommodating a greater number of sarcomeres in series, this architectural advantage may not be fully exploited during vertical jumping. 

An interesting finding of the present study was that none of the GM architectural variables were significantly associated with DJ-30 performance. Compared with the CMJ and SJ, the DJ represents a SSC task characterized by a shorter ground contact time and greater reliance on the rapid storage and release of elastic strain energy within the muscle-tendon unit [50]. Previous in vivo ultrasound studies have demonstrated that, in trained track-and-field athletes performing DJs from different heights, GM fascicles tend to remain relatively isometric while most of the length change occurs in the tendon, thereby enabling greater reactive power output and allowing more effective storage and return of elastic energy [49]. Accordingly, for the resistance- and plyometric-trained track and field athletes included in the present study, DJ performance may depend more on the mechanical properties of the tendon, neuromuscular coordination, and muscle–tendon interactions than on static GM architectural characteristics. This may partly explain the absence of associations between GM architectural variables and DJ-30 performance observed in the present study. Nevertheless, the relatively small sample size (*n* = 13) may have limited the statistical power to detect moderate associations. Therefore, these findings should be interpreted with caution and confirmed in studies with larger sample sizes.

Furthermore, the unadjusted analysis showed a positive relationship between Kvert and relative PF during the CMJ (*r* = 0.62, *p* = 0.02). This finding is consistent with that of Secomb et al. [13] who also reported a significant relationship between Kvert and CMJ PF in elite male surfing athletes (*r* = 0.60, *p* = 0.02). Together, these observations are consistent with previous work suggesting a relationship between lower-limb stiffness and dynamic lower-body performance [51]. Although the importance of lower-limb stiffness in supporting dynamic athletic performance has been previously recognized [3,49], to the best of our knowledge, the present study is the first to report this relationship in track and field athletes. Tendon stiffness is also thought to be important for dynamic performance [52] and has been shown to exhibit significant correlations with jump performance [51,52]. However, tendon stiffness was not measured in this study. Therefore, the contribution of tendon mechanical properties to the observed jump-performance relationships cannot be determined from the present data. Importantly, the relationship between calculated Kvert and CMJ relative PF should be interpreted with caution because PF is included in the calculation of Kvert, potentially inflating the observed association through mathematical coupling. Moreover, this association was attenuated after adjustment for body mass (*r* = 0.29, *p* = 0.35), sex (*r* = 0.32, *p* = 0.29), or both (*r* = 0.25, *p* = 0.44), indicating that the unadjusted relationship was not robust to covariate adjustment.

## 5. Limitation

First, the sample size was small (*n* = 13) and included athletes from different track and field disciplines, with an uneven sex distribution (11 males and 2 females). This limited statistical power and precision, and prevented meaningful subgroup comparisons by sex or event specialization. Given the multiple correlation analyses performed, none of the observed associations remained statistically significant after FDR correction, and the findings should therefore be regarded as exploratory and hypothesis-generating rather than confirmatory. Second, several potentially important determinants of jumping performance were not assessed, including muscle mass, neuromuscular activation, fiber-type composition, tendon mechanical properties, and individual movement strategies [53]. The observed associations between GM architecture, Kvert, and jump performance should therefore not be interpreted as independent effects of GM architecture alone. In addition, body mass may have contributed to associations involving absolute force and power, and although body-mass-adjusted partial correlations were performed, the small sample limited a more comprehensive evaluation of body-size effects. Third, GM fascicle length was estimated mathematically from MT and PA rather than measured directly, and extended field-of-view ultrasonography was not used. The estimated FL may therefore be subject to estimation error and may not fully capture regional variation in muscle architecture. The ICC values reflected measurement consistency but did not establish the accuracy or validity of the estimated FL. Fourth, a fixed 30-cm drop height was used for the DJ rather than an individualized optimal drop height. Although 30 cm has been used in previous studies, individual optimal drop heights may vary according to neuromuscular and anthropometric characteristics. The fixed-height protocol may therefore not have elicited maximal reactive performance in every athlete. Finally, the cross-sectional design precludes any conclusions regarding causality or directionality. In addition, the relationship between Kvert and CMJ relative PF should be interpreted with caution because peak force is a component of the Kvert calculation, and this association was not robust to covariate adjustment. Future studies with larger samples, longitudinal designs, and direct measurements of tendon and neuromuscular properties are needed to confirm and extend these findings. 

## 6. Conclusions

In unadjusted analyses, greater GM PA and MT showed nominal associations with selected force-, power-, and velocity-related outcomes during the SJ and CMJ, but none of these associations remained statistically significant after FDR correction. No significant associations were observed between GM architecture and DJ-30 performance. These findings provide preliminary evidence that GM architecture may relate differently to jump performance across tasks with different stretch-shortening cycle demands; however, this interpretation should be considered exploratory. Vertical stiffness also showed a nominal association with CMJ relative peak force, although this relationship should be interpreted cautiously given the potential for mathematical coupling and its attenuation after covariate adjustment. Further studies with larger samples and appropriate control of potential confounding factors are warranted to clarify the relationships between GM architecture, vertical stiffness, and different components of jumping performance.

## Figures and Tables

**Table 1:** Demographic and training characteristics of the collegiate track and field athletes according to event group.

Event	Sex	Age (years)	Height (m)	Body Mass (kg)	Training Age (yrs)
400-m sprint (*n* = 2)	Male 2	23.33 ± 1.15	1.80 ± 0.07	73.00 ± 6.00	5.00 ± 1.73
100-m sprint (*n* = 1)	Male 1	19.00 ± 0.00	1.83 ± 0.00	76.70 ± 0.00	7.00 ± 0.00
Hurdles (*n* = 4)	Male 3, Female 1	23.50 ± 2.65	1.84 ± 0.07	78.65 ± 12.54	8.25 ± 2.22
Jumping events (*n* = 6)	Male 4, Female 1	19.60 ± 1.34	1.80 ± 0.08	72.20 ± 8.10	5.60 ± 2.30
Total (*n* = 13)	Male 11, Female 2	21.62 ± 2.60	1.82 ± 0.06	74.72 ± 8.72	6.23 ± 2.55

**Table 2:** Descriptive statistics and within-session reliability of vertical jump performance variables in collegiate track and field athletes (*n* = 13).

	Track and Field Athletes	ICC (95% CI)	CV (%)
Countermovement jump			
Peak force (N)	1927.47 ± 264.73	0.98 (0.97, 0.99)	2.89
Relative peak force (N/kg)	26.09 ± 2.54	0.96 (0.92, 0.98)	3.54
Peak power (W)	4868.36 ± 832.45	0.99 (0.99, 0.99)	2.10
Relative peak power (W/kg)	64.24 ± 8.50	0.99 (0.97, 0.99)	2.25
Peak velocity (m·s^−1^)	3.07 ± 0.21	0.96 (0.92, 0.98)	3.17
Jump height (m)	0.45 ± 0.06	0.98 (0.97, 0.98)	3.65
Kvert (N·m^−1^)	6198.23 ± 1465.48	0.94 (0.86, 0.98)	4.30
Squat jump			
Peak force (N)	1997.59 ± 313.73	0.99 (0.99, 0.99)	1.57
Relative peak force (N/kg)	26.37 ± 3.05	0.99 (0.99, 0.99)	1.49
Peak power (W)	4666.75 ± 763.38	0.99 (0.98, 0.99)	1.20
Relative peak power (W/kg)	62.13 ± 7.49	0.97 (0.94, 0.99)	1.50
Peak velocity (m·s^−1^)	2.97 ± 0.38	0.98 (0.96, 0.99)	3.17
Jump height (m)	0.42 ± 0.12	0.98 (0.97, 0.98)	2.98
DJ-30 cm			
Peak force (N)	3958.98 ± 848.54	0.91 (0.83, 0.96)	4.77
Relative peak force (N/kg)	51.05 ± 9.80	0.87 (0.74, 0.95)	4.82
Peak power (W)	4943.64 ± 637.01	0.97 (0.93, 0.99)	1.96
Relative peak power (W/kg)	65.30 ± 4.90	0.90 (0.82, 0.96)	2.02
Jump height (m)	0.31 ± 0.06	0.95 (0.91, 0.98)	3.18
Ground contact time (ms)	194.08 ± 15.97	0.83 (0.64, 0.94)	6.27

Note: Values are presented as mean ± SD. ICC, intraclass correlation coefficient; CV (%), coefficient of variation; CMJ, countermovement jump; SJ, squat jump; DJ-30 cm, drop jump from 30 cm height; Kvert, vertical stiffness calculated from CMJ peak vertical ground reaction force divided by vertical displacement of the centre of mass.

**Table 3:** Pearson correlations between gastrocnemius medialis muscle architecture, vertical stiffness, and vertical jump performance variables.

Variable 1	Variable 2	Raw *r*	95% CI	Raw *p*	FDR *q*	*r*^2^ (%)
GM PA	SJ PF	0.66	0.18, 0.89	0.01	0.27	43.6
GM PA	CMJ PF	0.56	0.02, 0.85	0.04	0.27	31.4
GM PA	CMJ PP	0.62	0.10, 0.87	0.02	0.27	38.4
GM PA	CMJ height	0.55	0.01, 0.84	0.04	0.27	30.3
GM MT	CMJ PV	0.56	0.01, 0.85	0.04	0.27	31.4
GM MT	CMJ rPP	0.62	0.11, 0.87	0.02	0.27	38.4
Kvert	CMJ rPF	0.62	0.11, 0.87	0.02	0.27	38.4

Note: Only correlations reaching the nominal significance level (unadjusted *p* < 0.05) are presented. GM, gastrocnemius medialis; PA, pennation angle; MT, muscle thickness; CMJ, countermovement jump; SJ, squat jump; Kvert, vertical stiffness; PF, peak force; PP, peak power; rPP, relative peak power; PV, peak velocity; CI, confidence interval; FDR, false discovery rate; *q*, FDR-adjusted *p*-value; *r*, Pearson correlation coefficient; *r*^2^, coefficient of determination.

**Table 4:** Covariate-adjusted partial correlations for correlations with unadjusted *p* < 0.05 between GM muscle architecture, Kvert, and jump performance variables.

Variable 1	Variable 2	Unadjusted (*r*/*p*)	Partial (*r*/*p*) Adjusted for Body Mass	Partial (*r*/*p*) Adjusted for Sex	Partial (*r*/*p*) Adjusted for Body Mass and Sex
GM pennation	CMJ PF	0.56 (0.04)	0.45 (0.13)	0.34 (0.26)	0.34 (0.29)
GM pennation	CMJ PP	0.62 (0.02)	0.53 (0.07)	0.29 (0.34)	0.27 (0.41)
GM pennation	CMJ height	0.55 (0.04)	0.52 (0.08)	0.17 (0.59)	0.19 (0.56)
GM pennation	SJ PF	0.66 (0.01)	0.59 (0.04)	0.50 (0.09)	0.53 (0.09)
GM thickness	CMJ rPP	0.62 (0.02)	0.62 (0.03)	0.40 (0.18)	0.32 (0.32)
GM thickness	CMJ PV	0.56 (0.04)	0.57 (0.05)	0.18 (0.56)	0.13 (0.69)
Kvert	CMJ rPF	0.62 (0.02)	0.29 (0.35)	0.32 (0.29)	0.25 (0.44)

Note: Only correlations with unadjusted *p* < 0.05 in the primary correlation analysis were included in the sensitivity analyses. Unadjusted *r* represents the Pearson correlation coefficient without covariate adjustment. Partial *r* represents the partial correlation coefficient after adjustment for the covariate(s) specified in the corresponding column. GM, gastrocnemius medialis; PA, pennation angle; MT, muscle thickness; Kvert, vertical stiffness; CMJ, countermovement jump; SJ, squat jump; PF, peak force; rPF, relative peak force; PP, peak power; rPP, relative peak power; PV, peak velocity.

## Data Availability

The original contributions presented in this study are included in the article. Further inquiries can be directed to the corresponding author.

## Abbreviations

The following abbreviations are used in this manuscript:

GM	Gastrocnemius Medialis
SJ	Squat Jump
CMJ	Countermovement Jump
DJ	Drop Jump
Kvert	Vertical Stiffness
PF	Peak Force
PP	Peak Power
MT	Muscle Thickness
PA	Pennation Angle
FL	Fascicle Length
VL	Vastus Lateralis
LG	Lateral Gastrocnemius
SSC	Stretch-Shortening Cycle
COM	Centre of Mass
ICC	Intraclass Correlation Coefficients
CV	Coefficients of Variation
PV	Peak Velocity
SD	Standard Deviation
GCT	Ground Contact Time
FDR	False Discovery Rate
rPF	Relative Peak Force
rPP	Relative Peak Power
RSI	Reactive Strength Index

## Appendix A

### Appendix A.1

**Table A1:** Unadjusted and Benjamini–Hochberg FDR-adjusted correlations between GM muscle architecture, vertical stiffness, and jump performance

GM Muscle Architectural Variable	Outcome Variable	Correlation Coefficient	95% CI	Raw *p*	FDR-Adjusted *q*	Shared Variance (*r*^2^, %)
GM pennation	CMJ PF	0.56	0.02, 0.85	0.04	0.27	31.4
	CMJ rPF	0.33	−0.27, 0.74	0.27	0.68	10.9
	CMJ PP	0.62	0.10, 0.87	0.02	0.27	38.4
	CMJ rPP	0.50	−0.07, 0.82	0.08	0.29	25.0
	CMJ PV	0.52	−0.04, 0.83	0.06	0.27	27.0
	CMJ height	0.55	0.009, 0.84	0.04	0.27	30.3
	Vertical stiffness	0.21	−0.38, 0.83	0.48	0.81	4.4
	SJ PF	0.66	0.18, 0.89	0.01	0.27	43.6
	SJ rPF	0.52	−0.03, 0.83	0.06	0.27	27.0
	SJ PP	0.54	−0.01, 0.84	0.05	0.27	29.2
	SJ rPP	0.50	−0.05, 0.82	0.07	0.27	25.0
	SJ PV	0.31	−0.28, 0.73	0.30	0.73	9.6
	SJ height	0.33	−0.26, 0.74	0.26	0.68	10.9
	DJ-30 PF	0.16	−0.42, 0.65	0.58	0.81	2.6
	DJ-30 *r*PF	−0.05	−0.58, 0.51	0.86	0.92	0.3
	DJ-30 PP	0.20	−0.39, 0.67	0.50	0.81	4.0
	DJ-30 *r*PP	−0.17	−0.66, 0.42	0.57	0.81	2.9
	DJ-30 height	0.47	−0.10, 0.81	0.10	0.34	22.1
	DJ-GTC	0.21	−0.38, 0.68	0.47	0.81	4.4
GM thickness	CMJ PF	0.29	−0.30, 0.72	0.33	0.74	8.4
	CMJ rPF	0.32	−0.27, 0.74	0.27	0.68	10.2
	CMJ PP	0.51	−0.05, 0.83	0.07	0.27	26.0
	CMJ rPP	0.62	0.11, 0.87	0.02	0.27	38.4
	CMJ PV	0.56	0.01, 0.85	0.04	0.27	31.4
	CMJ height	0.54	−0.01, 0.84	0.05	0.27	29.2
	Vertical stiffness	0.25	−0.34, 0.70	0.39	0.81	6.3
	SJ PF	0.21	−0.38, 0.68	0.48	0.81	4.4
	SJ rPF	0.21	−0.37, 0.68	0.46	0.81	4.4
	SJ PP	0.43	−0.15, 0.79	0.13	0.42	18.5
	SJ rPP	0.51	−0.04, 0.83	0.06	0.27	26.0
	SJ PV	0.11	−0.46, 0.62	0.70	0.85	1.2
	SJ height	0.09	−0.48, 0.61	0.77	0.89	0.8
	DJ-30 PF	−0.12	−0.63, 0.45	0.68	0.84	1.4
	DJ-30 *r*PF	−0.21	−0.68, 0.38	0.48	0.81	4.4
	DJ-30 PP	0.29	−0.31, 0.72	0.33	0.74	8.4
	DJ-30 *r*PP	0.39	−0.20, 0.77	0.18	0.55	15.2
	DJ-30 height	0.20	−0.38, 0.68	0.49	0.81	4.0
	DJ-GTC	0.05	−0.50, 0.59	0.84	0.92	0.3
GM length	CMJ PF	−0.19	−0.67, 0.40	0.52	0.81	3.6
	CMJ rPF	0.01	−0.53, 0.56	0.95	0.95	0.0
	CMJ PP	−0.03	−0.57, 0.53	0.92	0.94	0.1
	CMJ rPP	0.16	−0.42, 0.65	0.59	0.81	2.6
	CMJ PV	0.10	−0.47, 0.62	0.72	0.85	1.0
	CMJ height	0.05	−0.51, 0.58	0.86	0.92	0.3
	Vertical stiffness	0.04	−0.52, 0.58	0.89	0.92	0.2
	SJ PF	−0.35	−0.75, 0.24	0.23	0.67	12.3
	SJ rPF	−0.23	−0.69, 0.36	0.43	0.81	5.3
	SJ PP	−0.02	−0.57, 0.53	0.92	0.94	0.0
	SJ rPP	0.07	−0.49, 0.60	0.80	0.91	0.5
	SJ PV	−0.15	−0.64, 0.43	0.62	0.82	2.3
	SJ height	−0.17	−0.66, 0.42	0.57	0.81	2.9
	DJ-30 PF	−0.24	−0.70, 0.35	0.42	0.81	5.8
	DJ-30 *r*PF	−0.14	−0.64, 0.43	0.62	0.82	2.0
	DJ-30 PP	0.12	−0.45, 0.63	0.67	0.84	1.4
	DJ-30 *r*PP	0.52	−0.03, 0.83	0.06	0.27	27.0
	DJ-30 height	−0.19	−0.67, 0.40	0.53	0.81	3.6
	DJ-GTC	−0.12	−0.63, 0.45	0.68	0.84	1.4
Kvert	CMJ rPF	0.62	0.11, 0.87	0.02	0.27	38.4

Note. MG, medial gastrocnemius; PF, peak force; rPF, relative peak force; PP, peak power; rPP, relative peak power; PV, peak velocity; GTC, ground contact time.

### Appendix A.2

**Table A2:** Personal best performances and primary events of the participating track and field athletes.

Subjects	Event Group	Personal Best Performance
1	400-m sprint	47.98 s
2	400-m sprint	49.00 s
3	100-m sprint	11.02 s
4	100-m hurdles	14.26 s
5	110-m hurdles	14.98 s
6	110-m hurdles	15.62 s
7	110-m hurdles	13.94 s
8	Long jump	7.76 m
9	Long jump	5.95 m
10	Long jump	7.24 m
11	Long jump	6.56 m
12	Long jump	6.97 m
13	Long jump	7.21 m
